# Serum Survivin Increases in Prolactinoma

**DOI:** 10.14740/jocmr2098w

**Published:** 2015-02-09

**Authors:** Fatma Dilek Dellal, Mutlu Niyazoglu, Suheyla Gorar, Esranur Ademoglu, Zehra Candan, Handan Bekdemir, Yalcin Hacioglu, Fatih Oner Kaya

**Affiliations:** a Department of Endocrinology, Ankara Training and Research Hospital, Ankara, Turkey; b Department of Endocrinology, Istanbul Training and Research Hospital, Istanbul, Turkey; c Department of Family Medicine, Istanbul Training and Research Hospital, Istanbul, Turkey; d Department of Internal Medicine, Istanbul Training and Research Hospital, Istanbul, Turkey

**Keywords:** Survivin, Prolactinoma, Microprolactinoma, Macroprolactinoma, Pituitary neoplasms

## Abstract

**Background:**

Prolactinoma is the most common adult pituitary adenoma. Survivin is a member of the family of inhibitors of apoptosis proteins. Its expression is observed in many tumors. Survivin expression has shown in prolactinoma tissue before but no study exists showing serum survivin level. The aim of the present study was to investigate serum survivin levels in patients with prolactinoma and demonstrate its value in diagnosis of the disease.

**Methods:**

The group of patients consisted of 25 women, aged from 17 to 51 years. As a control group, 21 healthy women, aged from 22 to 45 years were included. Twenty patients had microprolactinoma, while five patients had macroprolactinoma. All patients had received dopamine agonist treatment. Serum survivin levels were measured in all of the groups.

**Results:**

Survivin levels were significantly higher in prolactinoma patients compared to controls (19.04 (10 - 38) pg/mL; 15.05 (8 - 22) pg/mL; P = 0.042). There was no difference between microadenoma and macroadenoma patients in survivin levels (19.22 (10 - 38) pg/mL; 18.40 (16 - 22) pg/mL; P = 0.914). In correlation analysis, survivin was not correlated with other parameters.

**Conclusions:**

We consider that higher survivin levels might be a molecular marker predicting the presence of prolactinoma and may be useful for the diagnosis. But large-scale research is needed to clarify its role in diagnosis of prolactinoma patients.

## Introduction

Survivin, a member of the inhibitors of the apoptosis (IAPs) protein family, is encoded by BIRC5 gene located 17q25 in the human sequence. It is the smallest protein of the IAPs protein family, a 142 amino acid and a 16.5 kDa protein [1]. It has a single baculovirus IAP repeat (BIR) domain, which is responsible for protein recognition and interaction [2]. It regulates cell proliferation and apoptotic cell death while enhancing angiogenesis [2, 3].

Expression of survivin increases during embryogenesis; however it decreases after birth with the exception of thymus, hematopoietic progenitor cells, and basal epithelial cells of the colon [1]. Furthermore its formation was revealed in many of human neoplasms such as breast, stomach, non-small cell lung, colorectal, esophagus, ovary, pancreas, bladder, prostate, larynx, papillary thyroid, endometrium, cervix, glioma, neuroblastoma, melanoma, astrocytoma, and meningioma. Increased survivin expression is associated with clinicopathologic variables of aggressive disease and shows a strong correlation with shorter disease-free period in most studies [1, 4-16].

Pituitary adenomas are one of the most frequent intracranial neoplasms. Prolactinoma is the most common adult pituitary tumor accounting for 60% of functional pituitary adenomas [17]. Treatment is necessary in symptomatic patients and the primary therapeutic choice is medical therapy rather than surgery because the disease can be controlled, even cured with treatment of dopamine agonist in most cases [18, 19].

Increased survivin expression is shown in pituitary tumors but any study has not researched the survivin levels in plasma yet. In the current study, we aimed to determine serum survivin levels and evaluate its clinical significance at diagnosis of prolactinoma.

## Materials and Methods

Twenty-five female patients with prolactinoma with median age of 34 (17 - 51) years and 21 healthy female patients with a median age of 35 (22 - 45) years were included in the study. Patients were followed up at the Endocrinology outpatient clinic in the Ankara Training and Research Hospital. The patients were chosen among whose initial serum prolactin levels were > 250 ng/mL and the pituitary MRI showing adenoma [18]. Other functional pituitary tumors were excluded by dynamic hormone tests in all patients. The information of the tumor size was obtained from the last hypophysis MRI of patients. Twenty microprolactinoma patients and five macroprolactinoma patients were treated with appropriate doses of dopamine agonists. While 22 of them received cabergoline, three of them received bromocriptine. There was no tumor invasion to adjacent tissues in all patients. Having any other diseases (malignancy, diabetes mellitus, hypertension, hyperlipidemia, coronary artery disease, chronic liver or kidney diseases, gastrointestinal absorption problems, collagen tissue disease, and thyroid disease), and other functional or nonfunctional pituitary tumors were defined as exclusion criteria. All participants gave written informed consent and study was approved by local research ethics committee. The study was performed in accordance with Helsinki Declaration and Good Clinical Practice.

Body mass index (BMI) was calculated as the ratio of weight to the square of height (weight/heigh^2^ (kg/m^2^)).

Blood samples were collected after 12 h of fasting. Serum glucose, creatinine, alanine aminotransferase, thyroid stimulating hormone (TSH) and prolactin levels were measured. Biochemical parameters were studied using a Roche/Hitachi Modular autoanalyzer. In order to measure survivin levels, collected blood samples were centrifuged at 5,000 rpm/min after coagulation and stored at -80 °C until testing. Survivin was measured manually by EIA technique using Quantikine brand kits (Quantikine^®^ Survivin Elisa kit, R&D Systems, Minneapolis, MN, USA).

All data were analyzed statistically using SPSS Statistics version 17 (IBM). Differences in numerical data between two groups were evaluated by Mann-Whitney U test. Correlation of survivin with other variables was analyzed by Spearman’s rank test. Comparison of categorical variables was performed with Chi-square test and Fisher’s exact test. A P value less than 0.05 was considered statistically significant.

## Results

A total of 25 patients and 21 healthy controls were enrolled in the study. All patients were female. Median age was 34 years in the patient group (range, 17 - 51 years), while 35 years in the control group (range, 22 - 45 years). The median BMI was 27.4 kg/m^2^ in the patient group (range, 17.7 - 37.4 kg/m^2^), and 25.7 kg/m^2^ in the control group (range, 16.2 - 47.2 kg/m^2^). There was no difference between the patients and controls in age, and BMI (P = 0.921, P = 0.400, respectively). Both groups showed no difference regarding serum glucose, creatinine, alanine aminotransferase, and TSH levels. Median, minimum and maximum biochemical levels of parameters and P values in the patients and controls are as follows (glucose 87 (range, 77 - 132) mg/dL, 90 (range, 77 - 107) mg/dL, P = 0.408; creatinine 0.82 (range, 0.67 - 1.01) mg/dL, 0.84 (range, 0.67-1.07) mg/dL, P = 0.748; alanine aminotransferase 27 (range, 18 - 51) U/L, 32 (22 - 49) U/L; P = 0.169; TSH 1.84 (range 0.35 - 3.72) mIU/L, 1.69 (range 0.35 - 4.68) mIU/L, P = 0.332; respectively). The median prolactin levels were significantly lower in prolactinoma patients than in healthy controls (5.09 (range, 0.24 - 12.00) ng/mL, 17.38 (range, 10.94 - 24.05) ng/mL, P < 0.001) (Table 1). Survivin levels were significantly higher in the patients compared to controls (19.04 (10 - 39) pg/mL, 15.05 (8 - 22), P = 0.042). Median tumor size was 5 mm, and minimum and maximum sizes ranged between 2 and 26 mm in the patient group. After subgroup analysis, median sizes of microadenomas and macroadenomas were 5 (range, 2 - 7) and 12 (range, 11 - 26) mm. There was no difference between microadenoma and macroadenoma patients in survivin levels (19.22 (range, 10 - 38) pg/mL, 18.40 (range, 16 - 22) pg/mL; P = 0.914).

**Table 1:** Baseline Clinical Features of Prolactinoma Patients and Healthy Controls

	Patients group (n = 25)	Control group (n = 21)	P
Age (years)	34 (17 - 51)	35 (22 - 45)	0.921
BMI (kg/m^2^)	27.4 (17.7 - 37.4)	25.7 (16.2 - 47.2)	0.400
Glucose (mg/dL)	87 (77 - 132)	90 (77 - 107)	0.408
Creatinine (mg/dL)	0.82 (0.67 - 1.01)	0.84 (0.67 - 1.07)	0.748
ALT (U/L)	27 (18 - 51)	32 (22 - 49)	0.169
TSH (mIU/L)	1.84 (0.35 - 3.72)	1.69 (0.35 - 4.68)	0.332
Prolactin (ng/mL)	5.09 (0.24 - 12.00)	17.38 (10.94 - 24.05)	< 0.001*
Survivin (pg/mL)	19.04 (10 - 38)	15.05 (8 - 22)	0.042*

BMI: body mass index; ALT: alanin aminotranspherase; TSH: thyroid stimulating hormone. Mann-Whitney U test was used. Values are expressed as median (minimum-maximum). *Results are statistically significant (P < 0.05).

There was no correlation between survivin and other parameters in correlation analysis. Similarly, no correlation was found between tumor size and survivin levels (P = 0.565).

## Discussion

Pituitary tumors develop when the balance is disturbed between cell proliferation and cell death like other tumors. Activation of apoptosis suppressing signals has shown to be important in tumorigenesis and contributes to invasion and metastasis [18].

Survivin is undetectable in most adult tissues but its abundant expression was shown in most human tumoral cells [1, 6-16]. Retrospective clinical studies demonstrate that overexpression of survivin is correlated with increased proliferation, reduced apoptosis, and increased angiogenesis in colorectal cancer, whereas high levels of tumor survivin are associated with resistance to treatment and poor prognosis in multiple tumor types [20-25].

In addition to the studies demonstrating survivin expression in tissues, there are also some researches evaluating the serum levels of survivin in different cases [26-31]. Naumnik et al evaluated serum survivin, HMGB1, and VEGF in non-small cell lung cancer before and after four cycles of chemotherapy. Patients had higher HMGB1, VEGF levels while survivin levels were not different than healthy individuals. In conclusion, they pointed that HMGB1, VEGF, and survivin did not have any clinical significance in the prognosis of the survival time in lung cancer [30]. Goksel et al investigated the serum levels of her-2/neu and survivin in patients with early stage breast cancer. They found no difference in levels of serum her-2/neu and survivin between the breast cancer and the control group [26]. Bokarewa et al evaluated serum levels of survivin and antibodies against survivin in 131 rheumatoid arthritis patients. Results were related to joint erosivity at the time of sampling. Survivin levels were significantly higher in patients with destructive disease as compared with in RA patients displaying a non-erosive disease, whereas survivin antibodies led to a less aggressive course of the disease [31].

Survivin expression involving tissues of different pituitary adenomas was assessed in some studies. Most of the studies revealed higher survivin expression in pituitary tumors than in normal pituitary tissue [16, 32, 33]. Wasko et al investigated postoperative tissues of pituitary tumors of 43 patients and three normal pituitary tissues obtained at autopsy. Immunostaining was revealed nuclear localization of survivin in pituitary tumors as well as in normal pituitary tissue. Accumulation of survivin was less in normal pituitary than in tumoral tissues. Additionally, less intense immunohistochemical staining was observed in the cytoplasm of tumoral cells. They did not find any significant differences of accumulation between invasive and noninvasive tumors [16]. On the contrary, Zhang et al found significantly different expression rate in invasive and noninvasive tumors (89.7%, 40.7%, respectively) [32]. Jankowska et al found six-fold higher expression level of survivin expression in different pituitary tumors (acromegaly (n = 14), non-functioning pituitary tumor (n = 6), prolactinoma (n = 2) and corticotropinoma (n = 1)) than in normal pituitary tissue. It was not statistically significant difference between invasive and noninvasive tumors in terms of survivin level. Survivin was observed in the nuclei of pituitary tumors whereas it was restricted to small population of cells in normal pituitary tissue. They emphasized that overexpression of survivin is characteristic for pituitary tumors [33]. Contrary to the literature, Formasa et al did not find survivin expression in normal pituitary tissue in their study, including 47 pituitary adenomas (35 non-functioning, seven GH-secreting, three prolactinomas, and two ACTH-secreting tumors) and six normal controls [34]. Expression of survivin both in normal and neoplastic tissue suggests that the protein may play a regulative role in tissues during proliferation and it may also be a key factor in neoplastic formation of pituitary [1, 16, 23, 34, 35].

The primary therapeutic choice in prolactinoma is medical treatment with dopamine agonists that reduce the size of prolactinoma by inducing a reduction in cell volume, as well as causing perivascular fibrosis, partial cell necrosis, and reduce cellular DNA synthesis [36]. Moreover, dopamine induces apoptosis through the activation of terminal caspase, caspase-3 [37]. In the present study, the prolactin levels were significantly lower in patients with prolactinoma than healthy individuals because patients with prolactinoma were on dopamine agonist treatment. Although the effect of dopamine agonists on serum survivin levels is not known, it is impressive to find out statistically significant higher survivin levels in prolactinoma group than control. In this study, it is important that we showed persistently high levels of survivin in patients with prolactinoma despite normalized prolactin levels with medical treatment. In this respect, this is the first study evaluating serum survivin levels in prolactinoma patients under medical treatment. On the other hand, survivin was not correlated with prolactin levels and tumor size. In order to disclose any possible relationship, further prospective studies evaluating survivin and pituitary hormone levels and tumor size before and after treatment are required. By this way, possible relationship between pituitary tumors and survivin might be better clarified.

In conclusion, our data suggest that higher serum survivin levels might be a parameter for indicating prolactinoma. This study may guide to further researches.
